# Genome-wide blood DNA methylation alterations at regulatory elements and heterochromatic regions in monozygotic twins discordant for obesity and liver fat

**DOI:** 10.1186/s13148-015-0073-5

**Published:** 2015-04-02

**Authors:** Miina Ollikainen, Khadeeja Ismail, Kristina Gervin, Anjuska Kyllönen, Antti Hakkarainen, Jesper Lundbom, Elina A Järvinen, Jennifer R Harris, Nina Lundbom, Aila Rissanen, Robert Lyle, Kirsi H Pietiläinen, Jaakko Kaprio

**Affiliations:** Department of Public Health, University of Helsinki, Helsinki, Finland; Department of Medical Genetics, Oslo University Hospital and University of Oslo, Oslo, Norway; Obesity Research Unit, Research Programs Unit, Diabetes and Obesity, University of Helsinki, Helsinki, Finland; Department of Radiology, HUS Medical Imaging Center, Helsinki University Central Hospital, University of Helsinki, Helsinki, Finland; Division of Epidemiology, The Norwegian Institute of Public Health, Oslo, Norway; Department of Psychiatry, Helsinki University Central Hospital, Helsinki, Finland; Endocrinology, Abdominal Center, Helsinki University Central Hospital, Helsinki, Finland; Institute for Molecular Medicine FIMM, University of Helsinki, Helsinki, Finland; Department of Mental Health and Substance Abuse Services, National Institute for Health and Welfare, Helsinki, Finland

**Keywords:** DNA methylation, Epigenetics, Monozygotic twins, Obesity, Liver fat

## Abstract

**Background:**

The current epidemic of obesity and associated diseases calls for swift actions to better understand the mechanisms by which genetics and environmental factors affect metabolic health in humans. Monozygotic (MZ) twin pairs showing discordance for obesity suggest that epigenetic influences represent one such mechanism. We studied genome-wide leukocyte DNA methylation variation in 30 clinically healthy young adult MZ twin pairs discordant for body mass index (BMI; average within-pair BMI difference: 5.4 ± 2.0 kg/m^2^).

**Results:**

There were no differentially methylated cytosine-guanine (CpG) sites between the co-twins discordant for BMI. However, stratification of the twin pairs based on the level of liver fat accumulation revealed two epigenetically highly different groups. Significant DNA methylation differences (*n* = 1,236 CpG sites (CpGs)) between the co-twins were only observed if the heavier co-twins had excessive liver fat (*n* = 13 twin pairs). This unhealthy pattern of obesity was coupled with insulin resistance and low-grade inflammation. The differentially methylated CpGs included 23 genes known to be associated with obesity, liver fat, type 2 diabetes mellitus (T2DM) and metabolic syndrome, and potential novel metabolic genes. Differentially methylated CpG sites were overrepresented at promoters, insulators, and heterochromatic and repressed regions. Based on predictions by overlapping histone marks, repressed and weakly transcribed sites were significantly more often hypomethylated, whereas sites with strong enhancers and active promoters were hypermethylated. Further, significant clustering of differentially methylated genes in vitamin, amino acid, fatty acid, sulfur, and renin-angiotensin metabolism pathways was observed.

**Conclusions:**

The methylome in leukocytes is altered in obesity associated with metabolic disturbances, and our findings indicate several novel candidate genes and pathways in obesity and obesity-related complications.

**Electronic supplementary material:**

The online version of this article (doi:10.1186/s13148-015-0073-5) contains supplementary material, which is available to authorized users.

## Background

Obesity is associated with an increased risk for metabolic disorders, in particular type 2 diabetes mellitus (T2DM). However, the rate at which metabolic disturbances become clinically apparent varies. Approximately 30% of obese individuals appear metabolically healthy [1]. These individuals are insulin sensitive and have normal liver fat and visceral fat content, and their adipose tissue remains free of inflammation and mitochondrial dysfunction [1,2]. The causes of the variability in health-related responses to excess weight are poorly understood. Some of the variation may be due to genetic background, lifestyle, and other environmental factors. The development of high levels of liver fat predicts other metabolic complications, and nonalcoholic fatty liver disease is closely associated with obesity in many, but not all obese individuals [3]. However, the genetic and environmental factors are difficult to disentangle, and joint genetic and environmental processes underlying the development of metabolic consequences of obesity have not been studied.

Epigenetics, as a potential link between environmental exposures and gene activity, is an ideal approach to unravel the complex etiology of obesity and related comorbidities [4]. As epigenetic mechanisms react to different environmental factors, including nutrients [5-7], environmental components (such as chemicals, for example, from tobacco [8-10]), and metabolic states [11-13], in a tissue-specific manner, epigenetic studies may especially benefit characterization of early disease progression.

Despite the large number of epigenetic studies of obesity and metabolic diseases using animal models, there are few epigenetic studies of obesity in humans. Most of the human studies have explored the DNA methylation status of a few previously identified genes known to affect obesity [7,14,15] or obesity-associated traits [16]. To identify novel genes and pathways related to obesity and obesity-induced complications, hypothesis-generating epigenome-wide association studies (EWAS) are needed. Two previous studies using the HumanMethylation27 BeadChip with 27,000 cytosine-guanine sites (CpGs), primarily targeting gene promoters and CpG islands (CGIs), examined blood leukocytes of obese and lean adolescents [17,18]. Wang et al. [6] discovered two obesity-associated inflammatory genes (*UBASH3A* and *TRIM3*), and Almen et al. [18] identified 20 CpGs differentially methylated between the groups. A larger study looking at the impact of body mass index (BMI) on DNA methylation in different tissues using the HumanMethylation 450 BeadChip by Dick et al. found five probes correlated with BMI, three of which were in the intron of *HIF3A* [19]. Recent papers also show that specific DNA methylation profiles in blood [20] and liver [21] may provide a link between aging and obesity. None of the obesity-associated CpGs were common to all studies, which may be due to differences between the study populations, diverse genetic backgrounds, or heterogeneous metabolic phenotypes between different BMI categories. Importantly, these studies provide evidence that obesity is associated with DNA methylation changes in blood leukocytes. One recent paper has addressed a question whether obesity-associated metabolic syndrome differs from obesity without metabolic complications by studying adipose tissue. Guenard et al. identified over 3,000 genes and 41 pathways differentially methylated between the two groups [22].

Genome-wide methylation studies are greatly enhanced by the usage of samples of trait-discordant monozygotic (MZ) twins. MZ twin pairs have the same genomic sequence, and the study design thus controls for the genetic diversity that has encumbered previous studies comparing obese and lean groups. In addition to being genetically identical (excluding rare somatic mutations [23-25] and structural variations [26-28]), MZ twins are matched for many confounding factors (for example, age, sex, family background). Thus, the co-twin control design is ideal when identifying epigenetic changes induced by lifestyle and acquired obesity.

The aim of this study was to identify DNA methylation marks associated with acquired obesity with or without metabolic dysregulation and thereby to identify potential epigenetic biomarkers for unhealthy obesity. To do this, we studied genome-wide DNA methylation patterns and associated chromatin states in 30 extremely rare, clinically healthy young adult MZ twin pairs discordant for BMI, identified from population-based twin cohort studies comprising ten birth cohorts (*n* = 5,200 pairs). Detailed phenotyping for adiposity and metabolic status enabled further stratification into two metabolically distinct subgroups characterized by either elevated or normal liver fat in the heavy co-twins. The heavy co-twins with elevated liver fat present several blood metabolic alterations, such as increased amounts of glucose, lipid, cytokines, and coagulation factors [2]. As all of these are overproduced by the fatty liver [29], liver fat accumulation is an interesting intermediate phenotype linking obesity-related comorbidities and the search for novel epigenetic markers in blood DNA. This distinction revealed DNA methylation differences in the obesity subtype with elevated liver fat and associated metabolic disturbances and thereby shows that blood epigenetic profiling has a great potential to better characterize the obesity phenotype, and identify subjects most at risk for developing metabolic complications.

## Results

### Metabolic characterization of MZ twins

We studied MZ twin pairs discordant for BMI (delta BMI >3 kg/m^2^, range 3 to 10.13 kg/m^2^, *n* = 30) and concordant for BMI (delta BMI <1.6 kg/m^2^, range 0 to 1.6 kg/m^2^, *n* = 10). Among the BMI discordant pairs, the co-twins differed for subcutaneous, intra-abdominal and liver fat (*P* < 0.001, Additional file 1) and overall adiposity. No differences in these measures were observed in the BMI concordant pairs. In the discordant pairs, all measures of adiposity increased linearly for each unit increase of BMI, except for liver fat. In half of the discordant pairs for whom liver fat was measured (*n* = 12), both co-twins had low liver fat content (approximately 1% triglycerides from liver weight) whereas in the other half (*n* = 13), the heavier co-twins had an increase (on average 509%) in liver fat compared to the leaner co-twins (*P* = 0.0015, Additional file 1). Both liver fat concordant and discordant groups were equally discordant for BMI (mean BMI difference 5.9 kg/m^2^ for the liver fat concordant and 4.9 kg/m^2^ for the liver fat discordant, the distributions of BMI and liver fat discordances are shown in Additional file 2: Figure S1), as well as for subcutaneous and intra-abdominal fat (within-pair difference *P* < 0.01 for all, Additional file 1). In accordance with the known harmful metabolic effects of fatty liver, the pairs with large differences in liver fat were also highly discordant for several physiological parameters. In this group, the heavier co-twins had significantly higher fasting insulin and Homeostasis Model Assessment (HOMA) index, larger area under the curve (AUC) glucose and insulin during the oral glucose tolerance test (OGTT), higher low-density lipoprotein (LDL) and lower high-density lipoprotein (HDL) cholesterol, and higher high-sensitivity C-reactive protein (CRP) than their leaner co-twins (Additional file 1). They also had higher diastolic blood pressure. None of these differences, except for HDL, were observed in the group where the heavier co-twins had normal levels of liver fat. Based on these within-pair differences in the metabolic profiles, we hereafter refer to the pairs discordant for BMI but concordant for liver fat as the normal liver fat (nLF) group and the pairs discordant for both BMI and liver fat as the elevated liver fat (eLF) group. The within-pair differences in glucose tolerance and liver fat were different between these two groups (AUC glucose, *P* = 0.04; liver fat %, *P* = 3.85 × 10^−07^, Additional file 1). The heavy co-twins from the nLF and eLF groups differed for liver fat (*P* = 7.69 × 10^−07^) and fasting insulin (*P* = 0.02).

### Technical and biological variation

Genome-wide DNA methylation was measured in whole blood using the Infinium HumanMethylation 450 BeadChip (Illumina). We applied stringent quality control and filtering procedures to minimize technical variation. To test for the reliability and consistency of the data, we conducted three experiments. First, to ensure that the 450 BeadChip identifies genuine DNA methylation differences between MZ co-twins rather than artificial differences due to technical variation, we hybridized two technical replicates of six different samples on the bead chips. DNA methylation patterns of the technical replicates were highly similar, showing greater resemblance among the replicates (Euclidean distance, ED = 12.24) than within-twin pairs (ED = 13.86 for concordant and 15.75 for discordant pairs), and greater similarity within pairs than between-same sex unrelated individuals (ED = 21.65, Additional file 2: Figure S2). This indicates high quality of the data and that the within-pair methylation variation exceeds the technical variation. Second, to validate the within-pair DNA methylation differences, we performed EpiTYPER MassARRAY analysis on eight selected CpGs differentially methylated in the eLF group. Mean Pearson correlation of methylation differences at all CpGs between 450 BeadChip and EpiTYPER was 0.87, ranging from 0.65 to 0.96 (Additional file 2: Figure S3). This clearly shows that the observed differences in DNA methylation between MZ co-twins are genuine. Third, to validate the accuracy of the genome-wide DNA methylation mapping using the 450 BeadChip, we compared data generated by the Infinium platform and reduced representation bisulfite sequencing (RRBS) [30] from a different set of MZ twin pairs discordant for psoriasis (Gervin, K et al. manuscript in preparation). We observed a mean Pearson correlation of 0.96 across all CpGs covered by both methods (an Infinium probe and at least ten RRBS reads, *n* = 60,000) in ten representative samples (Additional file 2: Figure S4). This is in agreement with a previous study [31] and demonstrates the robustness of the Infinium technology.

### Estimated proportions of CD4+ cells and granulocytes differ within pairs

Our data are derived from peripheral whole blood, which comprises a mixture of different cell types. These cell types display different DNA methylation profiles, which can potentially confound the analyses if the proportions of the different cell types vary between cases and controls. Because the obese co-twins of the eLF group show low-grade inflammation as part of their metabolically disturbed obesity phenotype, and this is likely to affect the cell-type composition, we estimated the cell-type compositions in each sample. To do this, we applied a statistical algorithm predicting distributions of six blood cell types based on cell-specific 450 BeadChip methylation signatures [32]. These cell-type estimates revealed within-pair differences in granulocytes and CD4+ cells (false discovery rate (FDR) 0.02, Additional file 3) in the eLF group while the cell-type proportion estimates did not differ within the pairs in the nLF group. As the script for estimating the cell-type compositions is based on isolated cells from only 7 males [32], we investigated the accuracy of the estimated cell counts using 10 individuals from our twin cohort with both 450 K methylation data and differential blood cell counts. We found inconsistencies between the estimates and real cell counts as demonstrated by the moderate to low correlation coefficients (*r* = 0.64, *P* = 0.05 for lymphocytes; *r* = 0.74, *P* = 0.02 for granulocytes; and *r* = 0.24, *P* = 0.5 for monocytes). Due to these inconsistencies and, moreover, as our primary interest was to explore the DNA methylation fingerprint of ‘unhealthy obesity,’ characterized by high liver fat and several preclinical metabolic alterations (Additional file 1), and to find potential novel biomarkers that help in the detection of this complex phenotype, we decided not to correct the data with cell-type estimates prior to differential methylation analysis. By correcting we would have over-adjusted our data and missed important associations between DNA methylation and liver fat (see Additional file 4, and Additional file 2: Figure S5, for unadjusted *versus* cell-type adjusted data).

**Table 1:** **Enriched KEGG pathways**

**KEGG pathway**		**Score**	***P*** **value**	**FDR**	**CTEA score**	**CTEA** ***P*** **value**	**CTEA FDR**
BMI discordant							
4977	Vitamin digestion and absorption	−0.23	0.002	0.098	−0.23	0.002	0.065
4614	Renin-angiotensin system	−0.23	0.003	0.098	−0.22	0.009	0.195
eLF group							
260	Glycine, serine and threonine metabolism	−0.19	<0.001	<0.001	−0.16	0.001	0.033
340	Histidine metabolism	−0.13	0.002	0.052	−0.12	0.005	0.065
4977	Vitamin digestion and absorption	−0.19	0.003	0.052	−0.15	0.020	0.130
4614	Renin-angiotensin system	−0.22	0.004	0.052	−0.24	0.001	0.033
300	Lysine biosynthesis	−0.48	0.004	0.052	−0.44	0.003	0.065
780	Biotin metabolism	−0.61	0.006	0.056	−0.64	0.009	0.084
920	Sulfur metabolism	−0.29	0.006	0.056	−0.29	0.006	0.065
591	Linoleic acid metabolism	0.36	0.001	0.065	0.49	<0.001	<0.001
830	Retinol metabolism	−0.11	0.011	0.089	−0.19	0.005	0.065

1,000 permutations were performed with no probe number cutoffs. CTEA cell-type estimate adjusted.

### Obesity-associated DNA methylation differences are associated with elevated liver fat

**Figure 1:**
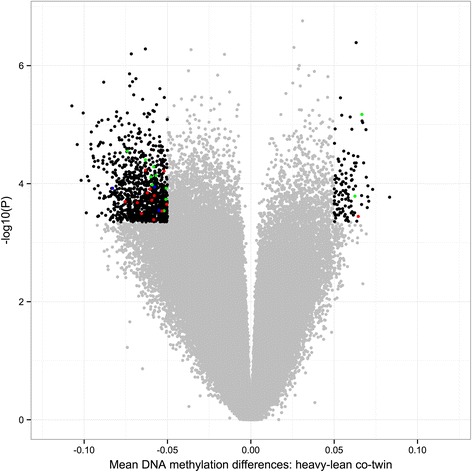
**Volcano plot of differences in DNA methylation between the discordant co-twins (** ***n*** **= 13 twin pairs) in the eLF group.** Each point represents a CpG site (*n* = 456,961) with mean within-pair differences in DNA methylation between co-twins on the *x*-axis and − log10 of the uncorrected *P* value from a paired test (moderated empirical Bayes) on the *y*-axis. Negative methylation differences indicate hypomethylation and positive differences hypermethylation in the heavy compared to the lean co-twins. Black dots represent significantly differentially methylated CpGs (*n* = 1,236, FDR <0.05, mean within-pair DNA methylation difference ≥5%); red dots represent CpGs located in genes genetically associated with obesity and obesity-associated traits (T2DM, liver fat, and MetS, *n* = 13, Additional file 5); green dots represent CpGs located in genes previously shown to be differentially methylated in obesity and T2DM (*n* = 11, Additional file 6); blue dots represent CpGs located in genes genetically and epigenetically associated to obesity and obesity-associated traits (*n* = 3, Additional files 5 and 6).

**Figure 2:**
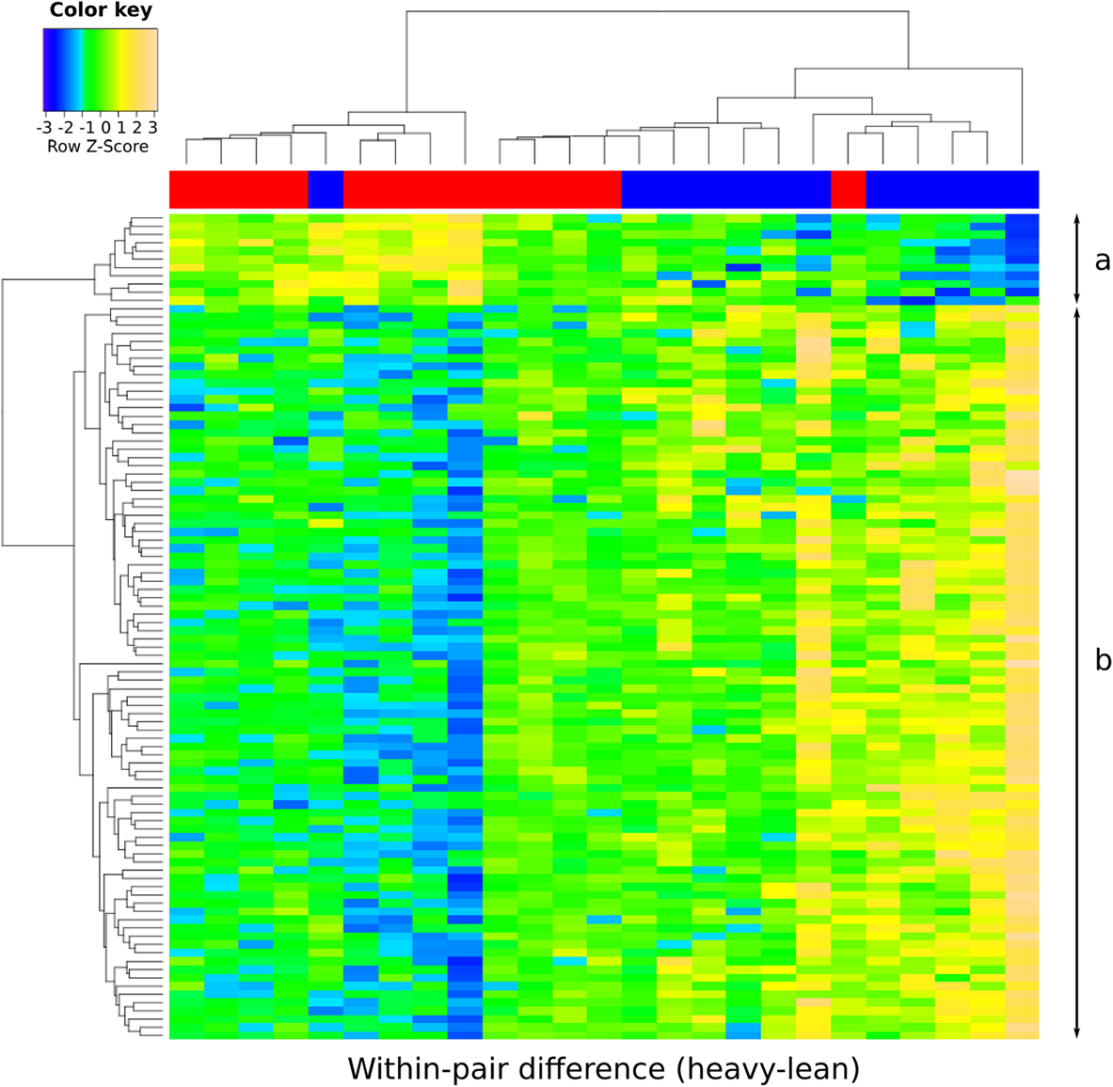
**Heat map of within-pair DNA methylation differences show clustering of the eLF and nLF groups.** Heat map of the within-pair DNA methylation differences (heavy-lean) at the top 100 most discordant CpGs (rows) identified in the eLF group show clustering of twin pairs (columns) in the eLF (red bar) and nLF (blue bar) groups. Color scale from blue to yellow represents the level and direction of within-pair methylation difference as *Z*-scores from negative towards positive values. **(a)** Heavy co-twins are more often hypermethylated relative to the lean co-twins in the eLF group and more often hypomethylated in the nLF group. **(b)** Heavy co-twins more often hypomethylated compared to the lean co-twins in the eLF group, and more often hypermethylated in the nLF group.

MZ co-twins were in general highly correlated for DNA methylation across all CpGs (mean = 0.996, range = 0.994 to 0.997). After correction for multiple testing and applying a biological relevance cutoff (mean within-pair methylation difference of ≥5%), none of the CpG sites were differentially methylated within the 30 BMI discordant twin pairs, nor in the 10 BMI concordant pairs (data not shown). Because the phenotypic characterization of these twins clearly identified two metabolically distinct groups, we sought to test the hypothesis that the eLF and nLF groups differ for their blood methylation profiles. While none of the CpGs were differentially methylated in the nLF group, 1,236 CpGs in 765 genes were differentially methylated in the eLF group (FDR <0.05, mean within-pair difference ≥5%, *n* = 13, range of mean methylation difference 0.05 to 0.11, Figure 1 and Additional file 4, and Additional file 2: Figure S6). In agreement with our hypothesis above, 1,042/1,236 CpGs showed different within-pair methylation discordances between the eLF and nLF groups (*P* < 0.05, Figure 2). This clearly demonstrates that the epigenetic dissimilarity is consistent with differences in metabolic parameters between the two groups.

### DNA methylation differences in obesity-associated genes

The genes located <10 Kb away from the SNPs or CpGs identified by genome-wide association studies (GWAS) [33-50] and EWAS [7,17,18,51-56] for obesity, liver fat, T2DM, and metabolic syndrome (MetS), which are covered by the 450 K bead chip (23 out of 247 genes retrieved from previous publications and from GWAS catalog www.ebi.ac.uk/gwas), were overrepresented among the 765 genes identified in the eLF group (Fisher’s exact test, *P* = 1.56 × 10^−4^, Figure 1, Additional files 5 and 6).

### Differentially methylated CpGs are overrepresented at promoters, insulators, and repressed states and are enriched for hypomethylation

**Figure 3:**
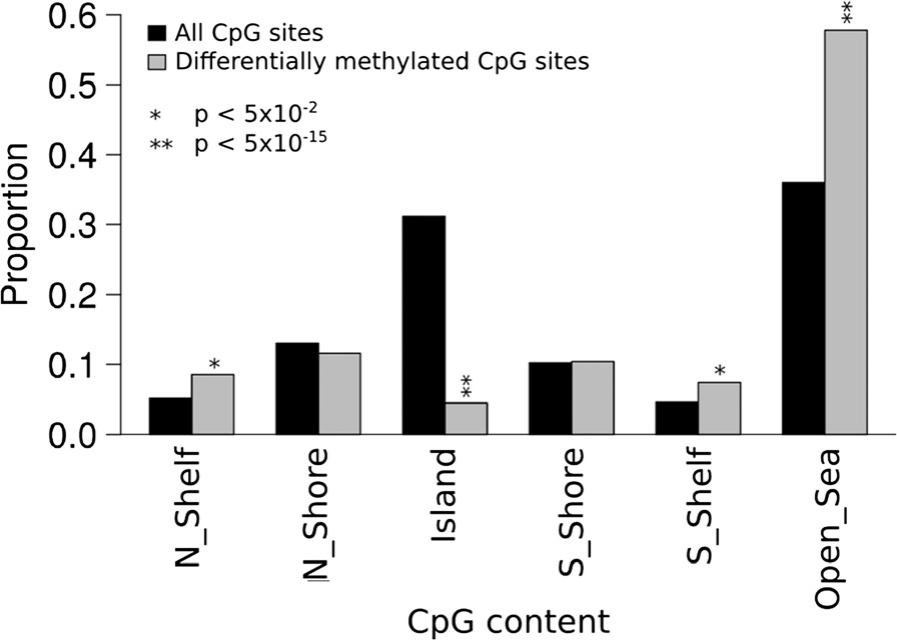
**CGIs underrepresented and open seas overrepresented among the differentially methylated CpGs.** Bar plot shows the proportions of the differentially methylated CpGs at CGIs, shores, shelves, and open sea and the *P* values denote which of the CpG categories are over- or underrepresented among the differentially methylated CpGs (*n* = 1,236) in the eLF group. Fisher’s exact test was used to generate *P* values for each group to see if they are under- or overrepresented among the 1,236 CpGs. Open sea, isolated CpGs outside any CGIs; shelves, 2 to 4 kb from CGI; CGI shores, <2 kb from CGI.

**Figure 4:**
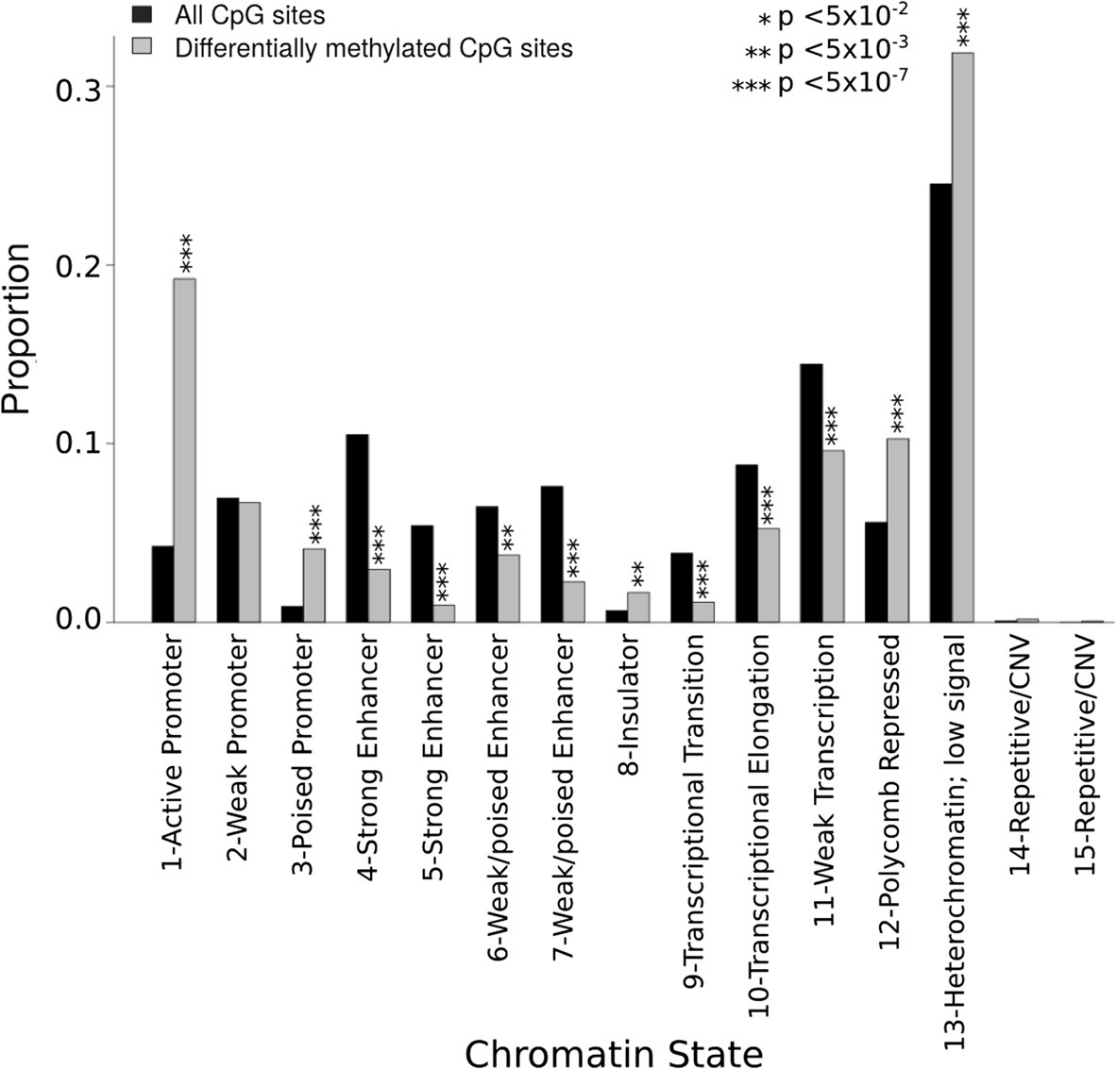
**Chromatin states at the differentially methylated CpGs.** Bar plot shows the proportions of 15 chromatin states using the Chromatin State Segmentation data from ENCODE/Broad Institute and which of the states are over- or underrepresented (Fisher’s exact test) among the differentially methylated CpGs (*n* = 1,236) in the eLF group. Chromatin states with identical names differ from each other by the frequency of each mark [58].

The genomic distribution of the differentially methylated CpGs in relation to CpG density (CGIs, shores, shelves, and open sea) in the eLF group was clearly different compared to the whole array CpG distribution (Figure 3). The differentially methylated CpGs were under-represented in CGIs and overrepresented in shelves and open seas. We also explored the location of the differentially methylated CpGs in relation to known and predicted functional elements in the genome (ChromHMM) by the use of ENCODE data from cell line GM12878 [57]. ChromHMM uses ChIP-seq data (CTCF and eight histone marks) to generate 15 chromatin states which are grouped to predict functional elements [58]. Based on the chromatin states, differentially methylated CpGs were overrepresented at active promoters, insulators, and within repressed and heterochromatic states and underrepresented at enhancers and transcribed sites (Figure 4).

**Figure 5:**
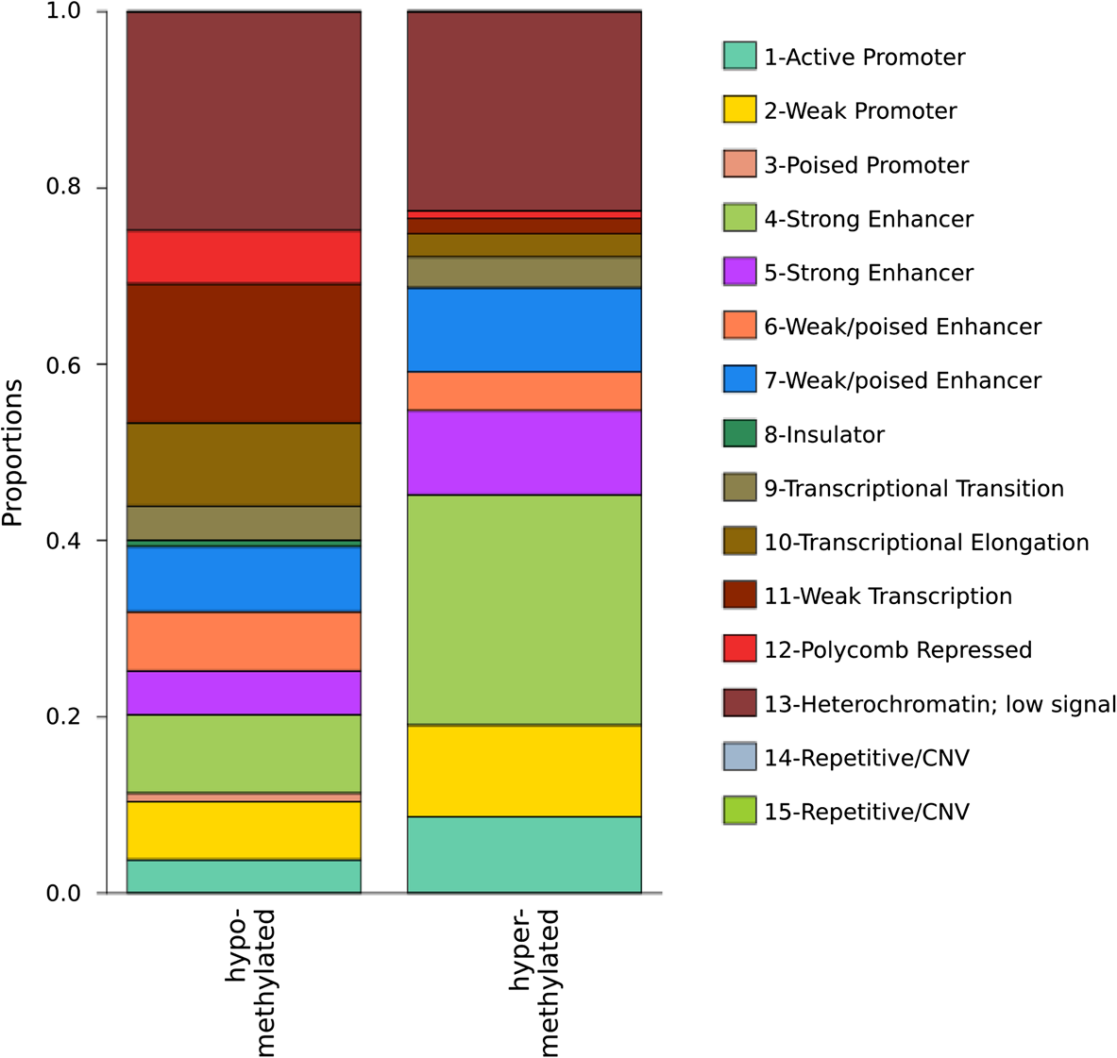
**Proportions of hypo- and hypermethylated CpGs in the heavy co-twins in relation to chromatin states.** The distributions of the hypo- and hypermethylated CpGs in relation to chromatin states differ greatly. Most of the hypomethylated CpGs were within heterochromatin (25%) whereas hypermethylated CpGs were most common at strong enhancers (26%).

Most of the differentially methylated CpGs were less methylated in the DNA from the heavy compared to the lean co-twins (1,121/1,236, 91%, *P* < 2.2 × 10^−16^, Figure 1) of the eLF group. In addition, based on the predicted chromatin states in the reference cell line, the functional genomic distribution of the hypo- and hypermethylated CpGs in the heavy co-twins showed distinct differences (Figure 5). Repressed chromatin and regions with weak transcription and transcription elongation were more often hypomethylated (*P* < 0.05) and active promoters and strong enhancers hypermethylated (*P* < 0.05) in the obese co-twins.

### Enrichment of gene sets and pathways relevant for obesity

Next, we performed gene set analysis (GSA) [59] to explore the potential of shared biologically relevant pathways among the obesity-associated methylation events. GSA of the BMI discordant group without stratification by liver fat discordance revealed only two enriched pathways (Table 1). Liver fat-stratified GSA did not reveal any pathways in the nLF group (data not shown); however, nine pathways showed enrichment in the eLF group (FDR <0.1, Table 1). Altogether eight of these pathways were less methylated in the heavy compared to their lean co-twins (Table 1). As shown in Additional file 2: Figure S7, vitamin- and amino acid-related pathways formed a linked network. To interpret the GSA data in the context of biological processes, pathways, and networks, the Core Analysis function in Ingenuity Pathways Analysis (IPA) (Ingenuity System Inc, USA) was performed for genes that map to the significant KEGG pathways from GSA (Additional file 7). Both GSA and IPA gave strong indications that the differentially methylated genes are involved with lipid, vitamin, and amino acid metabolism and immune and endocrine systems and liver dysfunction (Table 1).

### Metabolic measures lend support to the pathway findings

In light of the pathway results, we determined serum levels of linoleic acid, glycine, and histidine (Additional file 1). In the eLF group, the heavy co-twins had less linoleic acid than their lean co-twins. This was not seen in the nLF group where the relative circulating linoleic acid amount was similar in both co-twins. Also, glycine concentrations were reduced in the heavy co-twins of the eLF, but not of the nLF group. Histidine levels did not differ between the co-twins in either of the groups.

## Discussion

To our knowledge, this is the first comprehensive genome-wide leukocyte DNA methylation survey in MZ twin pairs discordant for BMI. Regardless of the within-pair discordance in BMI, the MZ twin pairs were highly similar for their methylation profiles, which is in line with previous DNA methylation studies using trait-discordant MZ twin pairs [60-67]. We show that DNA methylation profiles in blood leukocytes differ between BMI-discordant MZ co-twins only when high BMI is coupled with elevated liver fat and preclinical state metabolic disturbances (insulin resistance, low-grade inflammation, and dyslipidemia). This suggests that specific DNA methylation marks may identify obese individuals susceptible for the development of metabolic comorbidities. Altogether 91% of the differentially methylated CpGs were hypomethylated in the unhealthy obese as compared to their lean co-twins, including heterochromatic and repressed regions and promoters and insulators of both novel and known obesity-associated trait genes. The pathway analyses revealed clustering of the differentially methylated genes in vitamin (especially biotin and retinol), fatty acid, amino acid, and sulfur metabolism and to the renin-angiotensin system. These results suggest that the epigenetic signatures related to unhealthy obesity may involve nutritional factors, as well as point to possible new pathogenic routes to obesity-related diseases with implications for treatment.

Liver is a key metabolic regulator, and when fatty, it overproduces glucose, lipids, and inflammatory cytokines [68]. In turn, low liver fat has been suggested to be a hallmark of the so-called healthy obesity [69]. Here, as well as in our previous study [2], we showed that high liver fat is associated with several preclinical metabolic aberrations in obesity, including increased glucose, insulin, lipid, and CRP levels. With this in mind, it seems clear that the blood environment for the circulating leukocytes is different in subjects with high liver fat compared to those with low liver fat, that is, in those with or without metabolic aberrations. Because measurement of liver fat is laborious, blood epigenetic profiles together with metabolic surrogate markers may provide an attractive additional tool for the diagnosis and search for therapeutic targets in the future [29].

Among the differentially methylated genes in the eLF group, 23 have been previously found to be associated with obesity and obesity-associated traits in multiple GWAS meta-analyses and DNA methylation studies. For example, hypermethylation of *UBASH3A* and hypomethylation of *THADA* were found in the present as well as in previous studies [17,51].

We found that most of the differentially methylated CpGs were hypomethylated and associated with chromatin states marking important regulatory elements [52] and that differentially methylated CpGs were overrepresented at CGI shelves and open seas, and underrepresented at CGIs. This is in accordance with previous studies investigating disease-associated methylation patterns [70-73]. We also explored chromatin states at differentially methylated CpGs using ENCODE data and showed overrepresentation of promoters and insulators, suggesting that the methylation differences may have functional consequences by fine-tuning transcription of the associated genes. Unfortunately, no RNA is available from the study material to explore this hypothesis further.

We identified a large number of differentially methylated CpGs between co-twins highly discordant for liver fat and BMI. This was not a surprise as multiple genes in many pathways are likely to be differentially regulated in unhealthy obesity, and because we reported the ‘total’ within-pair methylation differences including both direct and indirect (via cell-type variation) obesity-associated methylation marks.

It is known that different cell subpopulations in peripheral blood may display different DNA methylation profiles. Consequently, there is a lot of discussion regarding the impact of cell-type heterogeneity on epigenetic studies performed on whole blood [74-81]. We acknowledge that our DNA methylation findings in the eLF group may be partly due to differences in cell-type proportions within pairs. Indeed, this may be the case in most studies comparing normal tissue to diseased tissue. Hence, in studies aiming to identify biomarkers for a disease state, adjusting for cell-type distributions could be over-adjustment leading to false negative findings. Even though co-twins from the eLF group differ for their estimates of granulocytes and CD4+ cells, the most prominent clinical parameters, liver fat, insulin resistance, and low grade inflammation, were only weakly correlated with the cell-type estimates. Regardless of the source of the observed methylation differences, our cell-type unadjusted methylation data distinguishing high BMI with and without a fatty liver may have a value for future development of diagnostic biomarker panels for early metabolic disturbances in obesity because such practical tools will be based on DNA from whole blood samples, not specific cell types. Further, the methylation profile of the eLF group may also serve as a useful tool in imputing the liver fat phenotype in epidemiological studies.

Like in any human study, we cannot exclude differences in all potential environmental factors within the twin pairs that may have an effect on DNA methylation. For example, recent alcohol intake has been shown to have genome-wide effects on blood DNA methylation [82]. Given the cross-sectional nature of our study, the direction of causality cannot be proven. We believe that only a minority of the observed methylation differences preceded obesity, but are mainly due to a complex mixture of different metabolic and clinical parameters (including blood cell-type heterogeneity), which are related to the complex phenotype differences between the lean and metabolically disturbed heavy co-twins characterized by elevated liver fat levels. However, we cannot preclude the possibility that some of the findings are indicative of processes that precede the onset of weight gain. Nevertheless, our main conclusion is that the observed epigenetic signature truly reflects the phenotype and characterizes the heavy twins in the eLF group.

## Conclusions

The present study shows that epigenetic profiling has a great potential to better characterize the obesity phenotype and identify subjects most at risk for developing metabolic complications. The metabolically disadvantaged obese MZ twins, with high liver fat, insulin resistance, inflammation, and dyslipidemia, were characterized by differential blood DNA CpG methylation in a number of novel and known obesity-associated genes when compared to the methylation levels of their lean counterparts. The pathways linked to the unhealthy obesity were related to vitamin, amino and fatty acid, renin-angiotensin, and sulfur metabolism. These results may harbor clues to the etiology, such as nutritional defects in the development of metabolic derangements. In addition to their potential role in diagnostics of the metabolic disturbances, the findings may help expedite the search for novel therapeutic targets for obesity.

## Methods

### Twin collection

The twin pairs were selected from two population-based longitudinal studies, FinnTwin16 and FinnTwin12, each consisting of five consecutive birth cohorts of Finnish twins [83]. Altogether 30 BMI discordant (within-pair difference (delta) in BMI ≥3 kg/m^2^) and 10 BMI concordant (delta BMI <1.6 kg/m^2^) MZ twin pairs were selected from the last follow-ups [2,84]. The range of the BMI of the lean co-twins was 19.7 to 40.6 kg/m^2^ and of the heavy co-twins was 24.2 to 48.6 kg/m^2^. Twins with concomitant somatic and psychiatric diseases or medications (except for oral contraceptives) were excluded.

The twin pairs (*n* = 40, 17 males, 23 premenopausal females) were 27 ± 3.3 (mean ± SD) years old. Their weight had been stable for at least 3 months prior to the study. Zygosity was confirmed by genotyping of ten informative genetic markers [85]. The subjects provided written informed consent. The protocol was designed and performed according to the principles of the Helsinki Declaration and was approved by the Ethics Committee of the Helsinki University Central Hospital.

### Phenotypic measurements

Weight and height were measured, after overnight fast, barefoot and in light clothing, to calculate BMI. Body composition was measured using whole body dual-energy x-ray absorptiometry (DEXA) [86]. Abdominal fat distribution and liver fat content were measured with a clinical magnetic resonance (MR) imager (1.5 Tesla, Avanto, Siemens, Erlangen, Germany) for MR imaging and MR spectroscopy [87]. MR images were analyzed using SliceOmatic v4.3 segmentation software and the results were expressed as total volumes of SAT and VAT. The liver spectra were analyzed with jMRUI v3.0 software [88] using the AMARES algorithm [89].

After 12-h overnight fast, subjects underwent a 75-g OGTT. Concentrations of plasma glucose were measured using the spectrophotometric hexokinase and glucose-6-phosphate dehydrogenase assay (Gluko-quant glucose/hexokinase, Roche Diagnostics, Tokyo, Japan) with a Hitachi Modular automatic analyzer (Hitachi, Tokyo, Japan), and serum insulin with time-resolved immunofluorometric assay (Perkin Elmer). Areas under the plasma glucose response curve (AUC glucose) and the serum insulin response curve (AUC insulin) were calculated from fasting, 30-, 60-, and 120-min glucose and insulin concentrations, with the trapezoid rule. The insulin resistance index (HOMA-IR) was calculated during OGTT according to Matthews et al. [90]. Total HDL cholesterol and triglyceride concentrations in serum were measured freshly by enzymatic methods (Roche Diagnostics Hitachi, Tokyo, Japan). LDL cholesterol concentrations were calculated using the Friedewald formula. Serum hsCRP was measured by particle-enhanced immunoturbidimetric assay (Cobas CRP(Latex)HS, Roche Diagnostics) on Modular automatic analyzer (Hitachi Ltd, Tokyo, Japan). Serum fatty acid and amino acid profiles were measured by proton NMR spectroscopy [91].

### DNA extraction and bisulfite conversion

High molecular weight DNA was extracted from whole blood using QIAamp DNA Mini kit (QIAGEN Nordic, Sollentuna, Sweden). Bisulfite conversion of DNA was completed using EZ-96 DNA Methylation-Gold Kit (Zymo Research, Irvine, CA, USA) according to the manufacturer’s instructions, and the co-twins were always converted on the same plate to minimize potential batch effects.

### DNA methylation analysis

DNA methylation status was assessed using the Infinium HumanMethylation 450 BeadChip, performed by the Microarray Consortium (Oslo, Norway) according to manufacturer’s instructions (Illumina, San Diego, CA, USA). The co-twins were always hybridized on the same chip.

All analyses were carried out using the R programming language (http://www.r-project.org/, v2.15) and Bioconductor (v2.10) [92]. The raw data was preprocessed using methylumi [93] and normalized using quantile normalization followed by beta-mixture quantile normalization (BMIQ). BMIQ effectively adjusts the data for the two different probe designs (Infinium type I and type II) on the array [94]. The ComBat function in the R package sva [95] was then used to correct for batch effects in the data. The data was filtered to remove probes with detection *P* values above 0.001 in any sample (5,372 probes), probes covering non-CpGs (3,063), and those mapping to X and Y chromosomes (11022). Further, Burrows-Wheeler Aligner (BWA) - short [96] - was used to identify probes that map to multiple locations in the genome (9,159 probes with >1 location), and all such probes were removed. This resulted in a final data set with 456,961 probes. Log2 ratios of methylated probe intensities to unmethylated probe intensities, the M-values, were then generated using functions in the R package lumi. Illumina Manifest was used for probe annotations.

Validation of within-pair differences at eight differentially methylated CpGs (in seven genes) in the eLF group was done by EpiTYPER MassARRAY (SEQUENOM Inc., Hamburg, Germany). PCR primers (Additional file 8) were designed using SEQUENOM’s EpiDesigner BETA (SEQUENOM Inc., Hamburg, Germany) tool (www.epidesigner.com). To reduce methylation variability introduced during PCR [97], triplicate amplifications of each sample were performed and pooled prior to MassARRAY analysis. PCR amplification was performed as in [98] and methylation status of each CpG was determined according to the manufacturer’s instructions.

### Statistical analysis

All differential methylation analyses were performed using M-values, and beta-values, ranging from 0 to 1 (0 to 100% methylation), were used to report the outcomes of the analyses. Statistical tests were conducted in R and Stata statistical software (release 12.0; Stata Corporation, College Station, TX). Comparisons of the clinical parameters within twin pairs were made by Wilcoxon signed-rank tests. Correlation of DNA methylation within discordant MZ twin pairs was computed as Pearson correlation and similarity as Euclidean distances (ED).

The level of discordance in technical replicates, twin pairs, and unrelated pairs was compared by using the mean of the ED in methylation within technical replicates of six samples, three randomly selected BMI concordant twin pairs and six randomly selected unrelated, same-sex individuals. Empirical cumulative distribution function (ECDF) plots were generated using means of the within-pair differences in the three pairs in each group. Probes containing SNPs (*n* = 59,892) were not included in computing the EDs or generating the ECDF plots. Furthermore, an algorithm developed by Houseman et al. [32] was utilized to determine the proportions of the white blood cell types (CD4^+^ and CD8^+^ T cells, CD56^+^ NK cells, B cells, monocytes, and granulocytes).

Differential methylation analysis was performed after adjusting the data for smoking, using empirical Bayes paired moderated *t* statistics implemented in the R package limma [99]. The raw *P* values from the paired tests were corrected for multiple testing using the Benjamini and Hochberg (BH) method. CpG sites with FDR <0.05 and within-pair methylation difference of ≥5% were called as differentially methylated. Moderated *t* statistics with BH correction was also used to compare the within-pair difference of twin pairs between groups to examine whether the identified within-pair methylation discordances were group specific.

The genomic distribution of the 1,236 differentially methylated CpGs, in relation to CGIs, was compared with the distribution of the CpGs in the whole data set. *P* values were computed using the Fisher’s exact test to determine over- or under-representation of the CpGs in relation to CGIs.

ENCODE data (ChromHMM on cell line GM12878, Broad Institute, Cambridge, MA, USA) was used to determine the chromatin state at each of the 1,236 CpGs by finding overlaps in the regions defined in the ENCODE data and the probe locations. *P* values were computed using Fisher’s exact test to determine if the differentially methylated CpGs over- or under-represented any of the chromatin states.

### Gene set and pathway analyses

The R package GSA was used to find the significance of predefined sets of CpGs, each set representing a pathway on KEGG. GSA was applied on the within-pair differences in methylation and run with 1,000 permutations. A *P* value cutoff of 0.05 and FDR cutoff of 0.1 were applied to obtain the list of significant pathways. GSA does not take into account the number of CpG sites on individual genes in a pathway; however, there was no obvious bias related to number of probes per genes (Additional file 2: Figure S8). The results give a list of pathways that need to be studied more closely. The genes in the significant KEGG pathways were further analyzed by IPA (Ingenuity Systems, Redwood City, CA, USA) to examine networks, functions, and associated diseases.

## Additional files

Additional file 1:
**Anthropometric and metabolic measures of the MZ twin pairs in the study.** Table with the characteristics of the study material. Additional file 2:
**A document with all supplementary figures (S1 to S8) and figure legends. Figure S1**: The distributions of BMI and liver fat discordances within pairs. **Figure S2**: Similarity of methylation between unrelated individuals, co-twins, and technical replicates. **Figure S3**: Validation of the within-pair DNA methylation differences. **Figure S4**: Comparisons of DNA methylation levels measured by Infinium 450 BeadChip and RRBS. **Figure S5**: QQ plots of observed *P* values from the within-pair methylation analysis of the eLF group twin pairs before and after correcting the data with estimated cell count proportions. **Figure S6**: Distribution of mean within-pair methylation differences and observed *P* values. **Figure S7**: Early onset liver fat-associated pathways form networks. **Figure S8**: Scatterplot of GSA results showing the mean number of probes per gene per pathway. Additional file 3:
**Within-pair differences of the estimated cell type proportions.** Table showing the within twin pair differences in the cell type estimates. Additional file 4:
**Differentially methylated CpGs in the eLF group.** Table listing the differentially methylated CpG sites with mean betas, mean delta betas, LogFC, and *P* values before and after cell type estimate adjustments. Additional file 5:
**Obesity and obesity-associated trait candidate genes identified by GWAS that are differentially methylated in the eLF group.** Table listing differentially methylated CpG sites that are located in obesity and obesity-associated trait candidate genes identified by GWAS. Additional file 6:
**Obesity and T2DM candidate genes identified by candidate gene DNA methylation or EWAS that are differentially methylated in the eLF group.** Table listing differentially methylated CpG sites replicating previously published obesity and T2DM associated methylation. Additional file 7:
**The top IPA networks among the significant pathways of the eLF group.** Table showing the enriched networks produced by IPA. Additional file 8:
**PCR primers for EpiTYPER MassARRAY analysis.** Table showing the primer sequences used in the validation assays.

## Abbreviations

BMI: body mass index
BMIQ: beta-mixture quantile normalization
CGIs: CpG islands
CpGs: CpG sites
ED: Euclidean distance
eLF group: elevated liver fat group
EWAS: epigenome-wide association studies
FDR: false discovery rate
GSA: gene set analysis
GWAS: genome-wide association studies
MetS: metabolic syndrome
MZ: monozygotic
nLF group: normal liver fat group
T2DM: type 2 diabetes mellitus
