# Supplementary material for: Genome-wide blood DNA methylation alterations at regulatory elements and heterochromatic regions in monozygotic twins discordant for obesity and liver fat
Source: Clin Epigenetics. 2015 Apr 2;7(1):39. doi: 10.1186/s13148-015-0073-5 (PMC4393626; doi:10.1186/s13148-015-0073-5)
Supplement: Additional file 2: — A document with all supplementary figures (S1 to S8) and figure legends. Figure S1: The distributions of BMI and liver fat discordances within pairs. Figure S2: Similarity of methylation between unrelated individuals, co-twins, and technical replicates. Figure S3: Validation of the within-pair DNA methylation differences. Figure S4: Comparisons of DNA methylation levels measured by Infinium 450 BeadChip and RRBS. Figure S5: QQ plots of observed P values from the within-pair methylation analysis of the eLF group twin pairs before and after correcting the data with estimated cell count proportions. Figure S6: Distribution of mean within-pair methylation differences and observed P values. Figure S7: Early onset liver fat-associated pathways form networks. Figure S8: Scatterplot of GSA results showing the mean number of probes per gene per pathway. [file 13148_2015_73_MOESM2_ESM.docx]

**Additional file 2.**

**Figure S1.** The distributions of BMI and liver fat discordances within twin pairs. **Figure S2:** Similarity of methylation between unrelated individuals, co-twins and technical replicates. **Figure S3:** Validation of the within-pair DNA methylation differences. **Figure S4:** Comparisons of DNA methylation levels measured by Infinium 450 BeadChip and RRBS. **Figure S5:** QQ plots of observed p-values from the within-pair methylation analysis of the eLF group twin pairs before and after correcting the data with estimated cell count proportions. **Figure S6:** Distribution of mean within pair methylation differences and observed p-values. **Figure S7:** Early onset liver fat-associated pathways form networks. **Figure S8:** Scatterplot of GSA results showing the mean number of probes per gene per pathway.


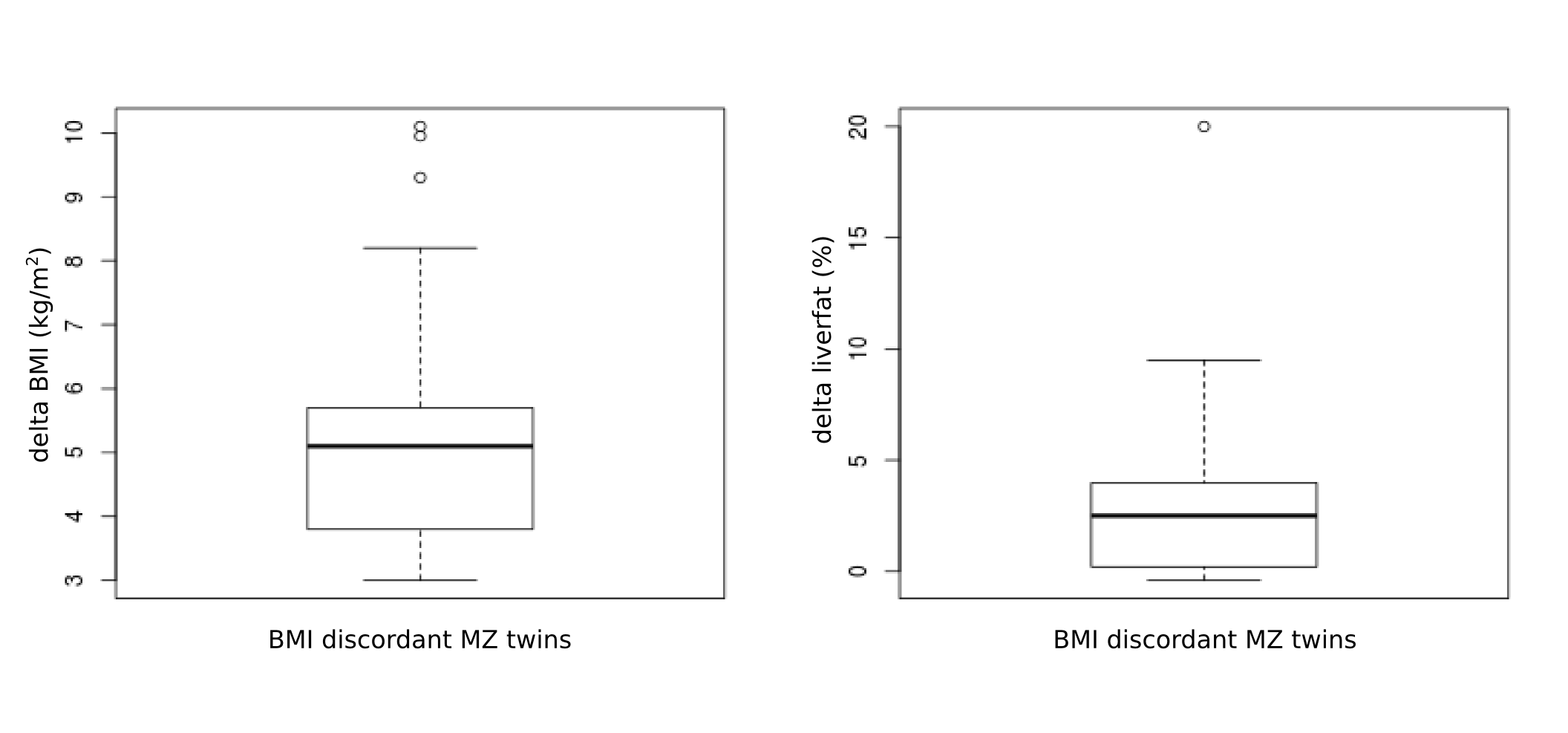


**Figure S1.** The distributions of BMI and liver fat discordances within twin pairs. All twin pairs with available BMI and liver fat data (n=25) are used in generating the box plots.


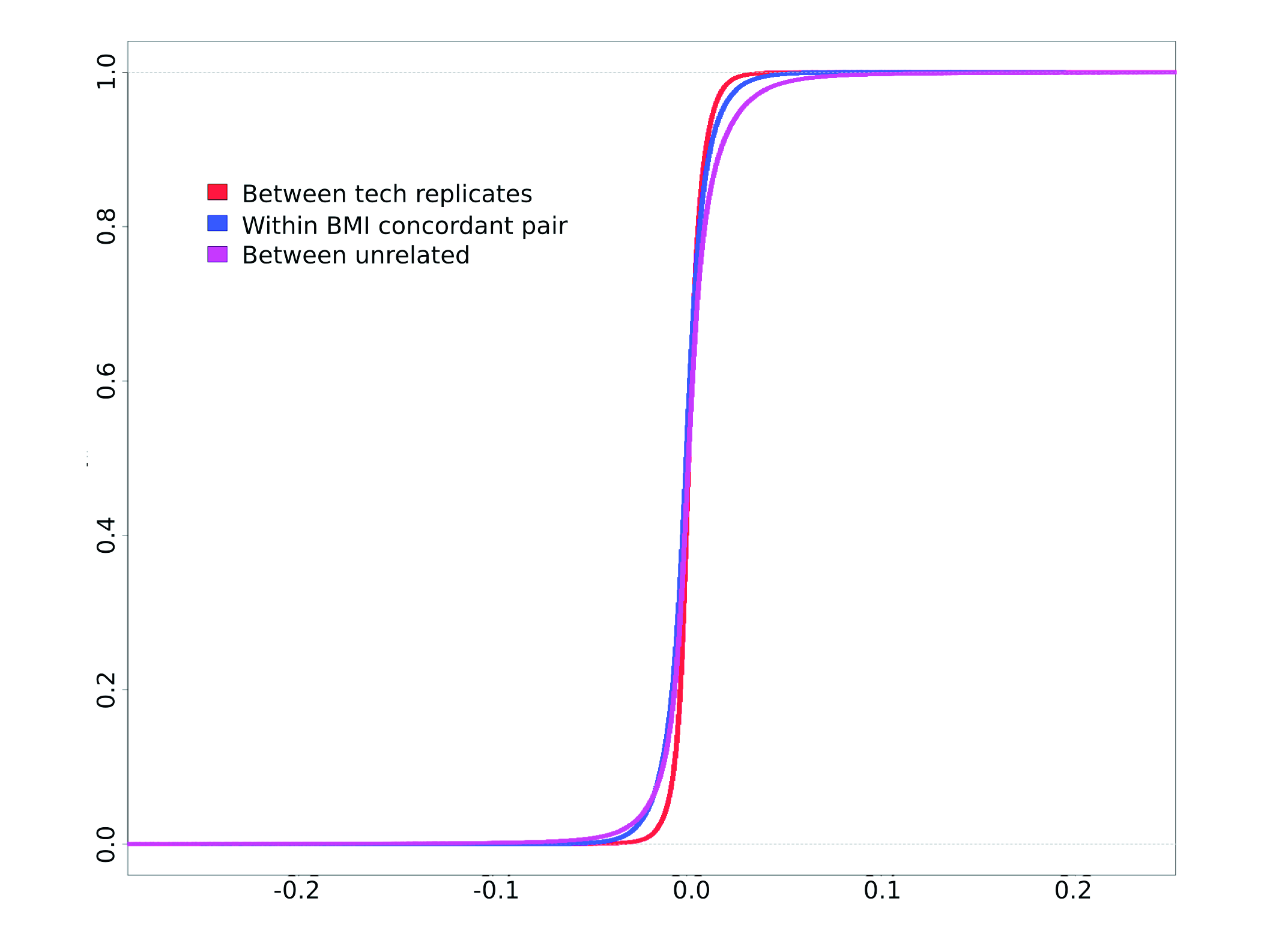


**Figure S2.** Similarity of methylation between unrelated individuals, co-twins and technical replicates. Methylation differences between unrelated individuals (purple), co-twins (blue) and technical replicates (red) were plotted as an empirical cumulative distribution function. Increasing similarity was observed from between comparisons of unrelated individuals to co-twins and technical replicates. Kolmogorov-Smirnov test showed that the samples have significantly different distribution (p<2.2x10^-16^). Euclidean Distance (ED) was also used as a measure for methylation differences between the samples. ED was 12.24 between technical replicates, 13.86 within concordant and 15.75 within discordant twin pairs and 21.65 between unrelated individuals. Two technical replicates of six samples, 3 MZ twin pairs and 6 same-sex unrelated individuals were used in plotting the graph.


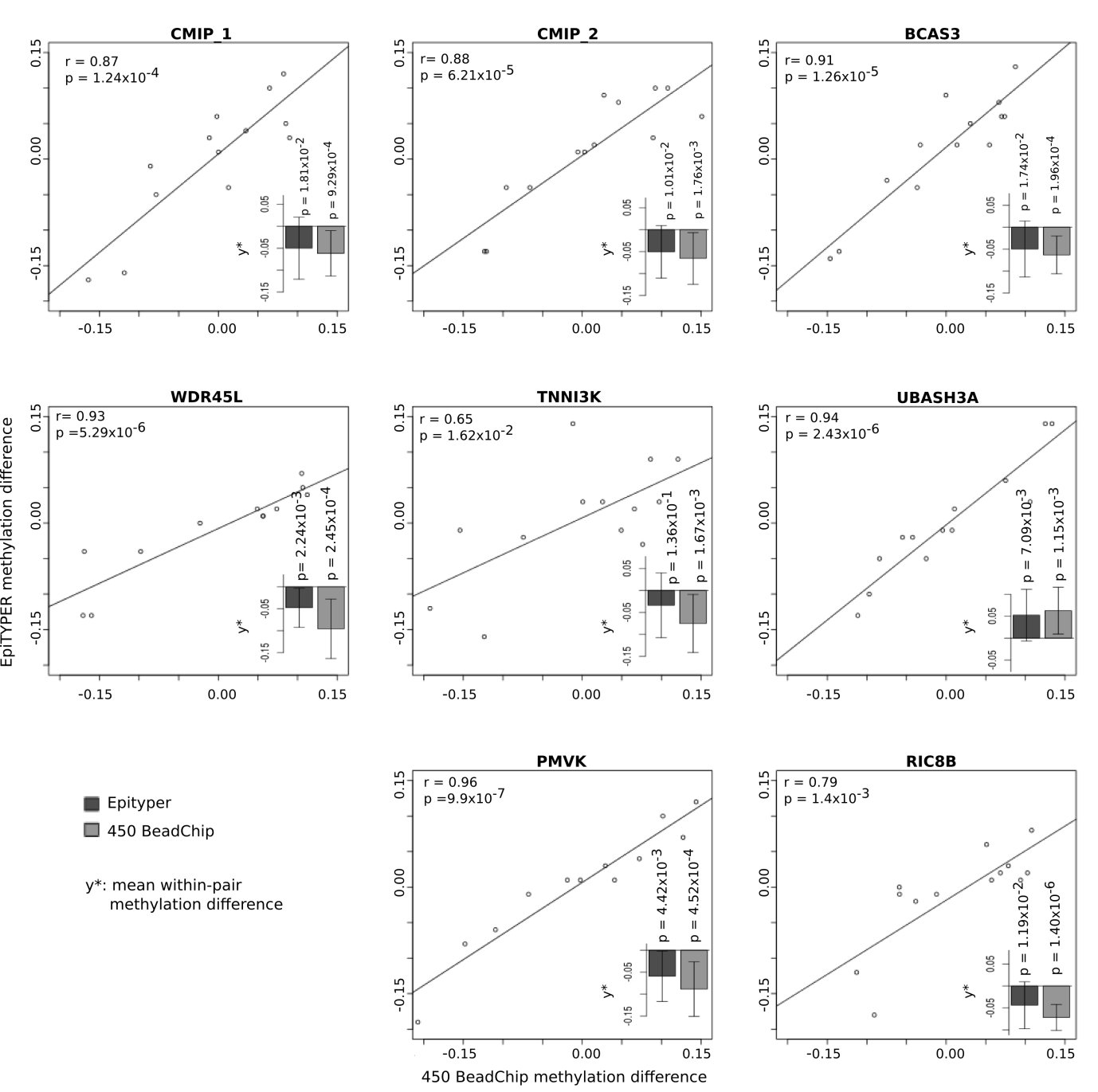


**Figure S3.** Validation of the within-pair DNA methylation differences. Scatter plots show the correlations of the within-pair DNA methylation differences between the 450 BeadChip and EpiTYPER MassARRAY data. The bar plots show mean ± sem methylation in the ‘lean’ and ‘obese’ twins for both the 450 BeadChip and EpiTYPER data. DNA methylation differences in eight CpGs significantly differentially methylated in the eLF group (n=13 pairs) by the 450 BeadChip data were selected based on hyper- and hypomethylated CpGs in our study (RIC8B, PMVK, WDR45L), CpGs replicating previously published obesity associated trait genes by DNA methylation (BCAS3, UBASH3A), and CpGs replicating previously published obesity associated trait genes by GWAS (CMIP, TNNI3K).


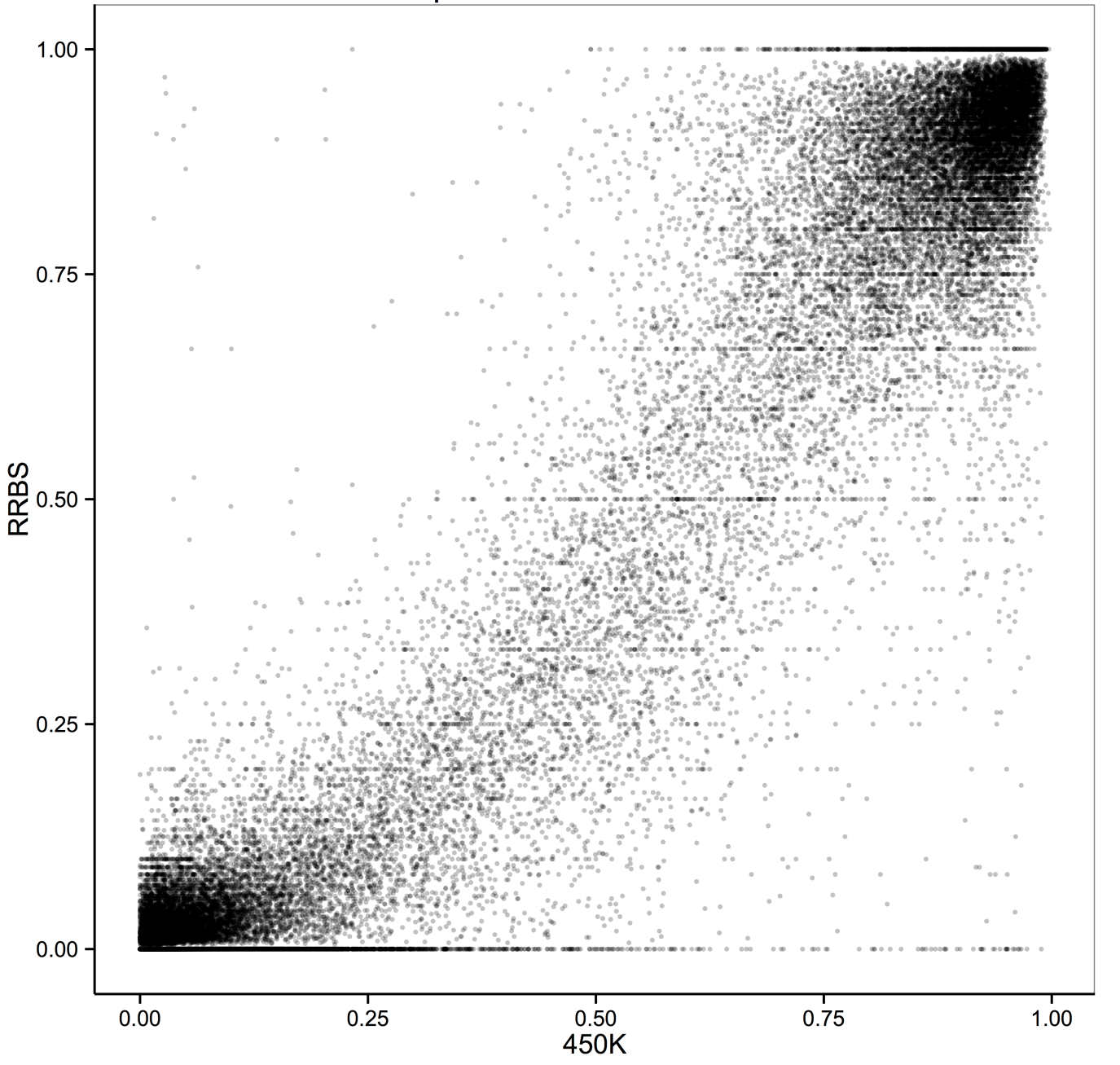


**Figure S4.** Comparisons of DNA methylation levels measured by Infinium 450 BeadChip and RRBS. Scatterplot of CpGs captured by Infinium 450 BeadChip probes and at least ten RRBS reads for one representative sample. Each spot represents a CpG site (n=63987).


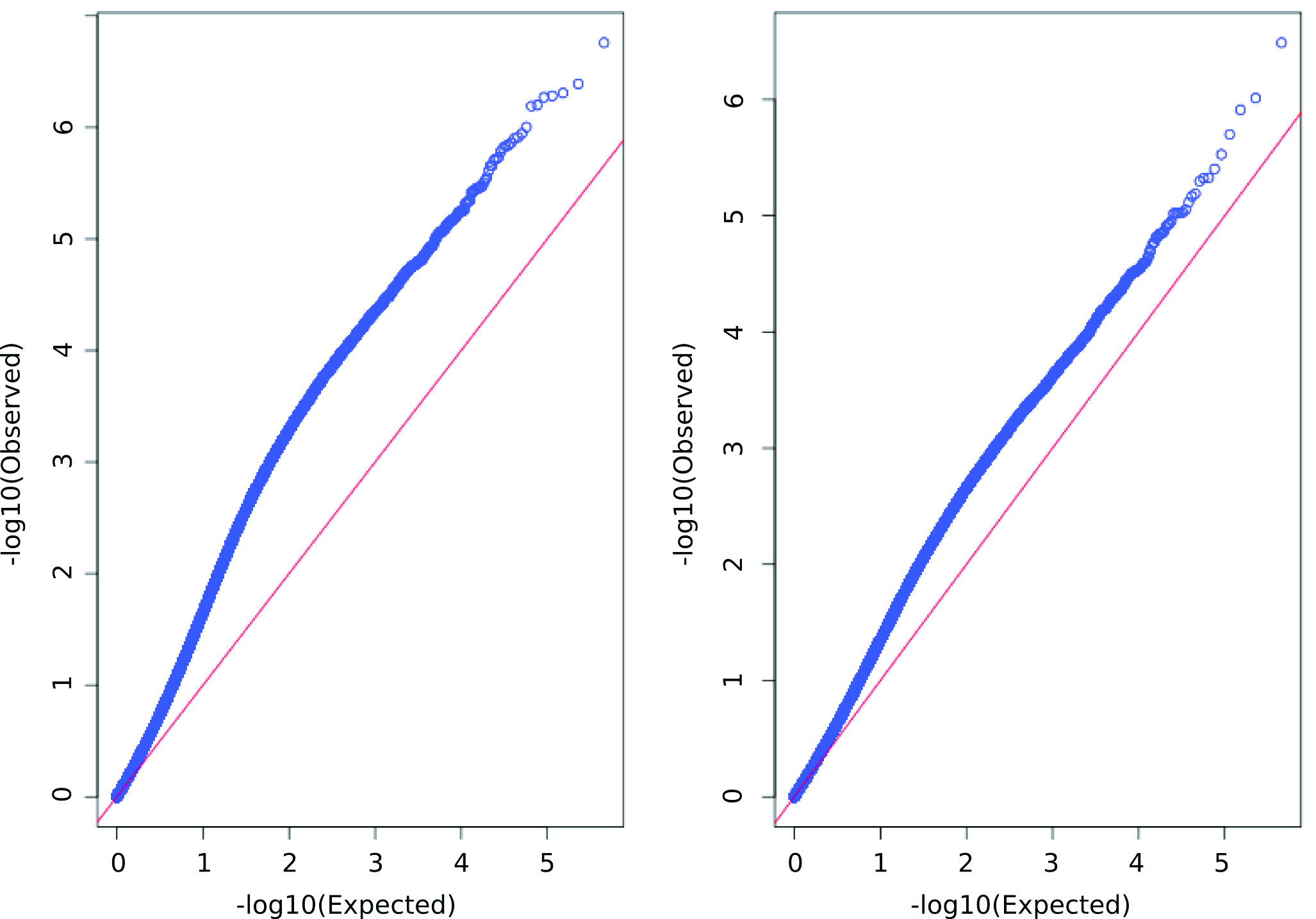


**Figure S5.** QQ plots of observed p-values from the within-pair methylation analysis of the eLF group twin pairs before and after correcting the data with estimated cell count proportions.


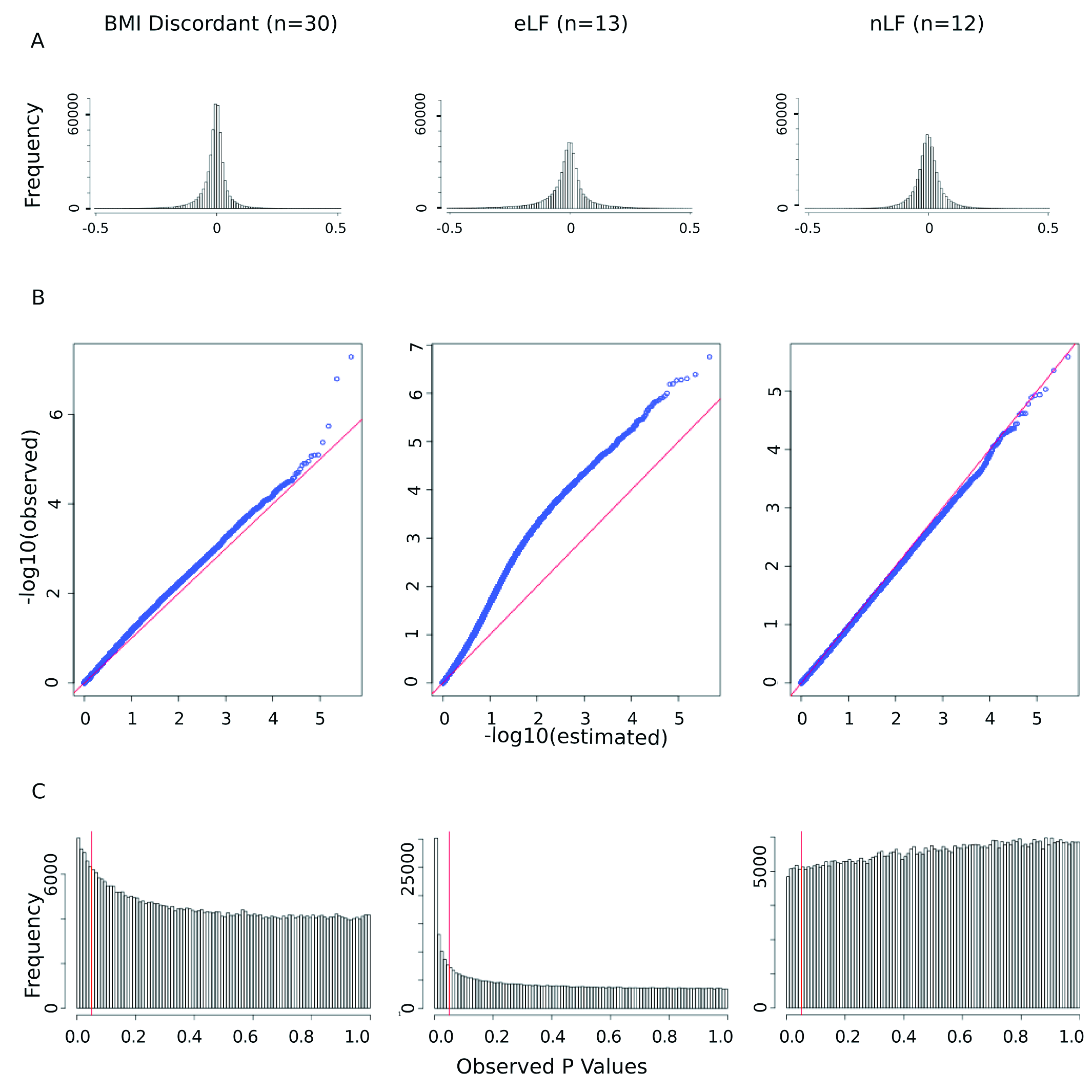


**Figure S6.** Distribution of mean within pair methylation differences and observed p-values. (A) Histograms show the distribution of the mean within pair methylation differences (mean delta betas) per CpG site for the BMI discordant, eLF and nLF twin pairs. The eLF group shows a smaller number of CpGs with mean delta betas close to zero compared to the BMI discordant and nLF groups. (B) QQ plots of observed p-values from the within-pair methylation analysis of BMI discordant, eLF and nLF twin pairs. P-values from the BMI discordant and nLF groups follow a uniform distribution (red line) as expected under the null hypothesis. P-values from the eLF group deviate from the uniform distribution indicating a large number of CpG sites having larger than expected within-pair differences. (C) The distribution of the observed p-values from the within-pair methylation analysis of BMI discordant, eLF and nLF twin pairs. The red line marks the p-value of 0.05. The eLF group shows a much higher frequency of low p values (<0.05) than the other two groups.


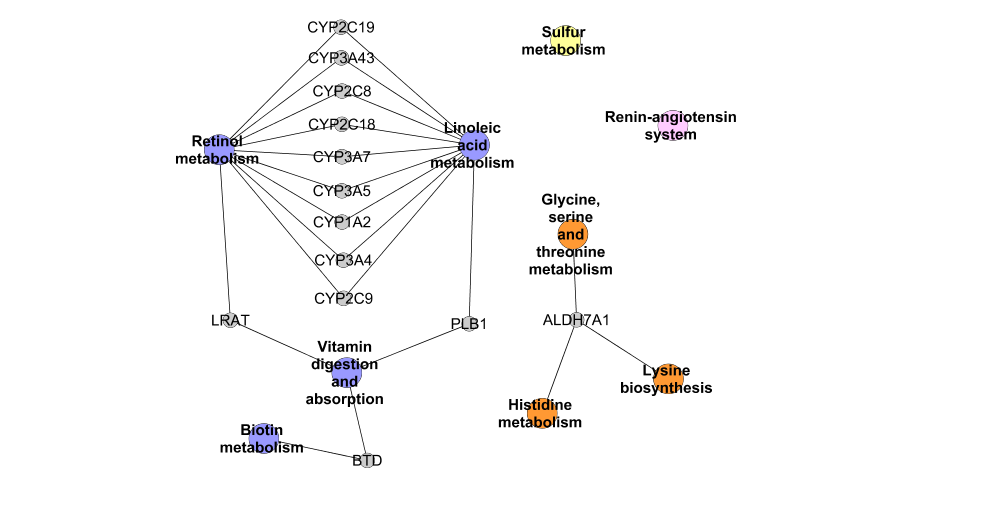


**Figure S7.** Early onset liver fat-associated pathways form networks. The significant KEGG pathways from the gene set analysis were linked to each other: Vitamin pathways and linked fatty acid pathway in blue, and amino acid pathways in orange. Renin-angiotensin system (pink) and Sulphur metabolism (yellow) do not link to the other pathways. Cytoscape was used to draw the network.


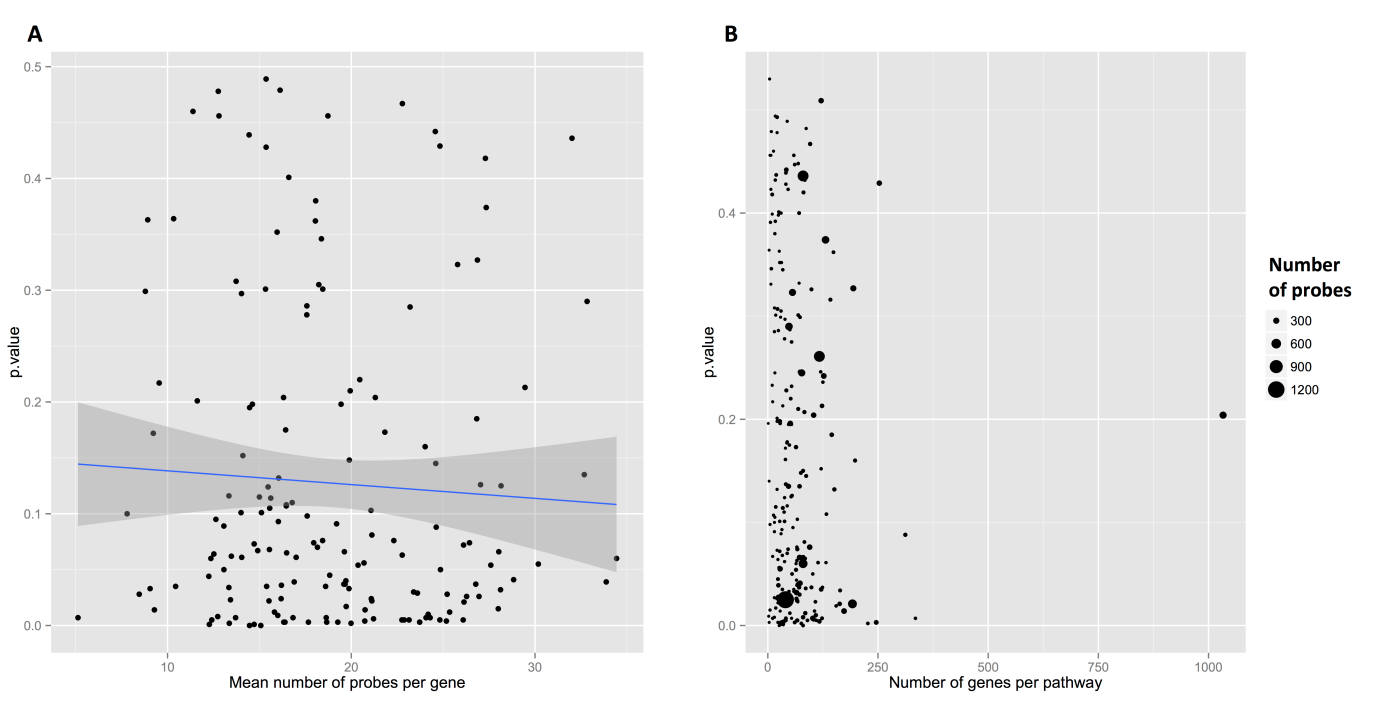


**Figure S8.** (A) Scatterplot of mean number of probes per gene per pathway (n=229, range= mean 5 to 55 probes per genes per pathway) included in the GSA on the x-axis against the p-value on the y-axis. Smoothening line represents local regression (method=linear) colored by time points. (B) Scatterplot (bubble chart) of the number of genes per pathway (n=5343 genes) on the x-axis against the p-value on the y-axis. The number of probes per gene in each pathway is mapped to the size of the points to illustrate the number of probes and genes per pathway.
